# Development and validation of an *in vitro* model system to study peripheral sensory neuron development and injury

**DOI:** 10.1038/s41598-018-34280-3

**Published:** 2018-10-29

**Authors:** Iwan Jones, Tushar Devanand Yelhekar, Rebecca Wiberg, Paul J. Kingham, Staffan Johansson, Mikael Wiberg, Leif Carlsson

**Affiliations:** 1 Umeå Center for Molecular Medicine (UCMM), Umeå University, Umeå, Sweden; 2 Laboratory of Neural Repair and Cellular Therapy, Department of Integrative Medical Biology (IMB), Umeå University, Umeå, Sweden; 3 Laboratory of Ion Channels and Neuronal Signaling, Department of Integrative Medical Biology (IMB), Umeå University, Umeå, Sweden; 4 Department of Surgical and Perioperative Sciences, Section of Hand and Plastic Surgery, Umeå University, Umeå, Sweden; 5 Present Address: McGovern Institute for Brain Research, Department of Brain and Cognitive Sciences, Massachusetts Institute of Technology (MIT), Cambridge, MA 02139 USA

## Abstract

The ability to discriminate between diverse types of sensation is mediated by heterogeneous populations of peripheral sensory neurons. Human peripheral sensory neurons are inaccessible for research and efforts to study their development and disease have been hampered by the availability of relevant model systems. The *in vitro* differentiation of peripheral sensory neurons from human embryonic stem cells therefore provides an attractive alternative since an unlimited source of biological material can be generated for studies that specifically address development and injury. The work presented in this study describes the derivation of peripheral sensory neurons from human embryonic stem cells using small molecule inhibitors. The differentiated neurons express canonical- and modality-specific peripheral sensory neuron markers with subsets exhibiting functional properties of human nociceptive neurons that include tetrodotoxin-resistant sodium currents and repetitive action potentials. Moreover, the derived cells associate with human donor Schwann cells and can be used as a model system to investigate the molecular mechanisms underlying neuronal death following peripheral nerve injury. The quick and efficient derivation of genetically diverse peripheral sensory neurons from human embryonic stem cells offers unlimited access to these specialised cell types and provides an invaluable *in vitro* model system for future studies.

## Introduction

The human peripheral nervous system (PNS) is a complex network of functionally distinct neurons that are organised into anatomically distinct ganglia. Mature dorsal root ganglia (DRG) are located adjacent to the spinal cord and are composed of heterogeneous populations of pseudounipolar peripheral sensory neurons that derive from delaminating neural crest cells in a step-wise hierarchical manner during development. Terminally differentiated sensory neurons are classified on the basis of their modality (nociceptors, proprioceptors and mechanoreceptors), axon diameter, myelination status, neurotrophic factor dependency and corresponding neurotrophic tyrosine receptor kinase (NTRK) expression signatures in addition to their innervation targets and neurotransmitter synthesis profiles^1,2^.

Human peripheral sensory neurons are inaccessible for research and much of our current understanding of sensory neuron diversity, development and disease derives from the use of animal models. Although rodent species faithfully recapitulate human peripheral sensory neuronal circuitry, most established models display large and heritable differences in modality-specific perception that correlates with genetic background. As such, some of the most critical developmental and disease related questions in human neurobiology have been difficult to address at the cellular and molecular level in animal models. These discrepancies therefore raise the question as to whether rodent species are faithful surrogates for modeling human peripheral sensory neuron development and disease^3–5^.

The *in vitro* differentiation of peripheral sensory neurons from human embryonic stem cells (hESCs) provides an attractive alternative to rodent models since an unlimited source of biological material can be generated for studies that specifically address human sensory neuron development and disease. Moreover, the *in vitro* derivation of peripheral neural networks is a critical goal in the regenerative medicine field since it underlies the future development of cell replacement therapies and novel analgesic treatments^6,7^. To this end, in the last decade several publications have described the derivation of peripheral sensory neurons from hESCs under a variety of differentiation regimes^8–14^. However, to fully exploit the potential of these hESC-derived peripheral sensory neuron models they must recapitulate the diversity of neuronal modalities found *in vivo* and the pathophysiological changes that underlie specific PNS injuries and diseases. This can only be accomplished by improving our current knowledge as to the molecular nature of the *in vitro* differentiation process in combination with in-depth molecular and functional analyses of the terminally differentiated neurons produced^15^. Moreover, the demonstration of experimental reproducibility by the routine use of these protocols in other laboratory environments will increase confidence in the stem cell community that these *in vitro* models are clinically useful and will ultimately result in the reduction of animal use in biomedical research^14^.

The work presented in this study describes how the use of small-molecule inhibitors is a robust method for deriving peripheral sensory neurons from hESCs. The resulting heterogeneous neuronal populations recapitulate several aspects of peripheral sensory neuron morphology and express established combinations of canonical- and modality-specific peripheral sensory neuron markers. Subsets of the derived cells also exhibit functional electrophysiological properties of human nociceptive neurons that include tetrodotoxin-resistant modalities in addition to associating with human donor Schwann cells in an *in vitro* co-culture system. Moreover, we show that the hESC-derived neurons can be used as a model system to investigate pathways of injury-induced cell death. Thus, the *in vitro* differentiated cells display several hallmarks of *bona fide* mature peripheral sensory neurons and provide an unlimited source of biological material for comparative studies that specifically address human sensory neuron development, injury and disease.

## Results

### Differentiation of peripheral sensory neurons from hESCs

**Figure 1:**
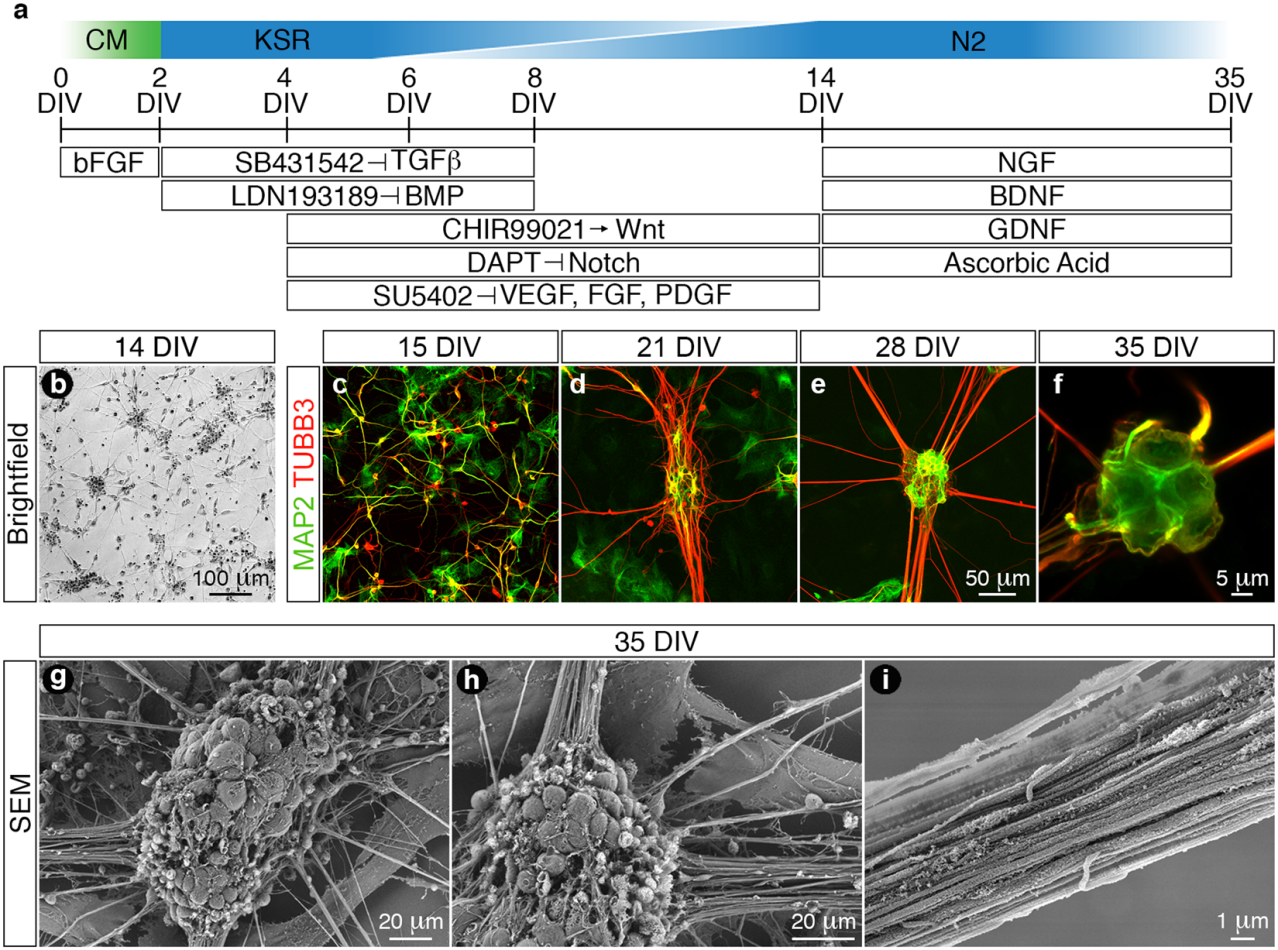
Differentiation of *in vitro* peripheral sensory neurons from hESCs. (**a**) Schematic diagram detailing the differentiation protocol employed in this study. hESCs were seeded as single cells and cultured in MEF conditioned medium until optimal confluence was reached (0–2 DIV). The hESCs were then differentiated using a combination of dual-SMAD inhibition and early WNT activation coupled with small-molecule inhibition of Notch, VEGF, FGF and PDGF signaling pathways (2–14 DIV). (**b**) Following completion of the differentiation phase (14 DIV) the derived cells exhibited typical immature neuronal morphology with each individual cell elaborating several neurites. (**c**–**f**) The cells (14 DIV) were replated in N2 medium supplemented with a defined neurotrophic factor cocktail (BDNF, GDNF, NGF and ascorbic acid) and time-course analyses using the pan-neuronal markers MAP2 and TUBB3 demonstrate that the differentiated neurons become migratory and transform over time from an arborised monolayer (**c**, 15 DIV) into neuronal ganglia that radially project axonal tracts (**f**, 35 DIV). (**g**–**i**) SEM analysis of the *in vitro* neuronal ganglia (35 DIV) demonstrates the intimate association of several neuronal somata of varying size classes (**g**) from which axons project radially away (**h**) and assemble into fasciculated nerve tracts (**i**). All images were derived from at least three independent differentiation experiments. Abbreviations: CM, conditioned medium; DIV, days *in vitro*; SEM, scanning electron microscopy. Scale bars: (**b**) 100 μm; (**c**–**e**) 50 μm; (**f**) 5 μm; (**g**–**h**) 20 μm; (**i**) 1 μm.

We generated peripheral sensory neurons from hESCs grown in conditioned medium by a combination of dual-SMAD inhibition and early WNT activation coupled with small-molecule inhibition of specific pathways including Notch, vascular endothelial growth factor (VEGF), fibroblast growth factor (FGF) and platelet-derived growth factor (PDGF) signaling (Fig. 1a)^16^. Following completion of the differentiation phase, we observed that a vast majority of the derived cells exhibited typical immature neuronal morphology with each individual cell elaborating several neurites (Fig. 1b). These immature cells were subsequently replated in N2 medium containing a defined neurotrophic factor cocktail including brain derived neurotrophic factor (BDNF), glial cell-derived neurotrophic factor (GDNF), nerve growth factor (NGF) and ascorbic acid. Sequential time-course analysis using the pan-neuronal markers neuronal-β-III-tubulin (TUBB3) and microtubule associated protein-2 (MAP2) demonstrated that the naïve neurons became highly migratory and transformed from arborised monolayers at 15 days *in vitro* (DIV) (Fig. 1c) into elaborate neuronal networks that were composed of distinct neuronal ganglia and radially projecting axonal tracts (21 to 35 DIV) (Fig. 1d–f). Furthermore, scanning electron microscopy demonstrated that the ganglia were comprised of coalesced neuronal somata of various diameters (Fig. 1g) and that the projecting axon bundles (Fig. 1h) assembled into fasciculated nerve tracts (Fig. 1i).

### Molecular characterisation of differentiated peripheral sensory neurons

**Figure 2:**
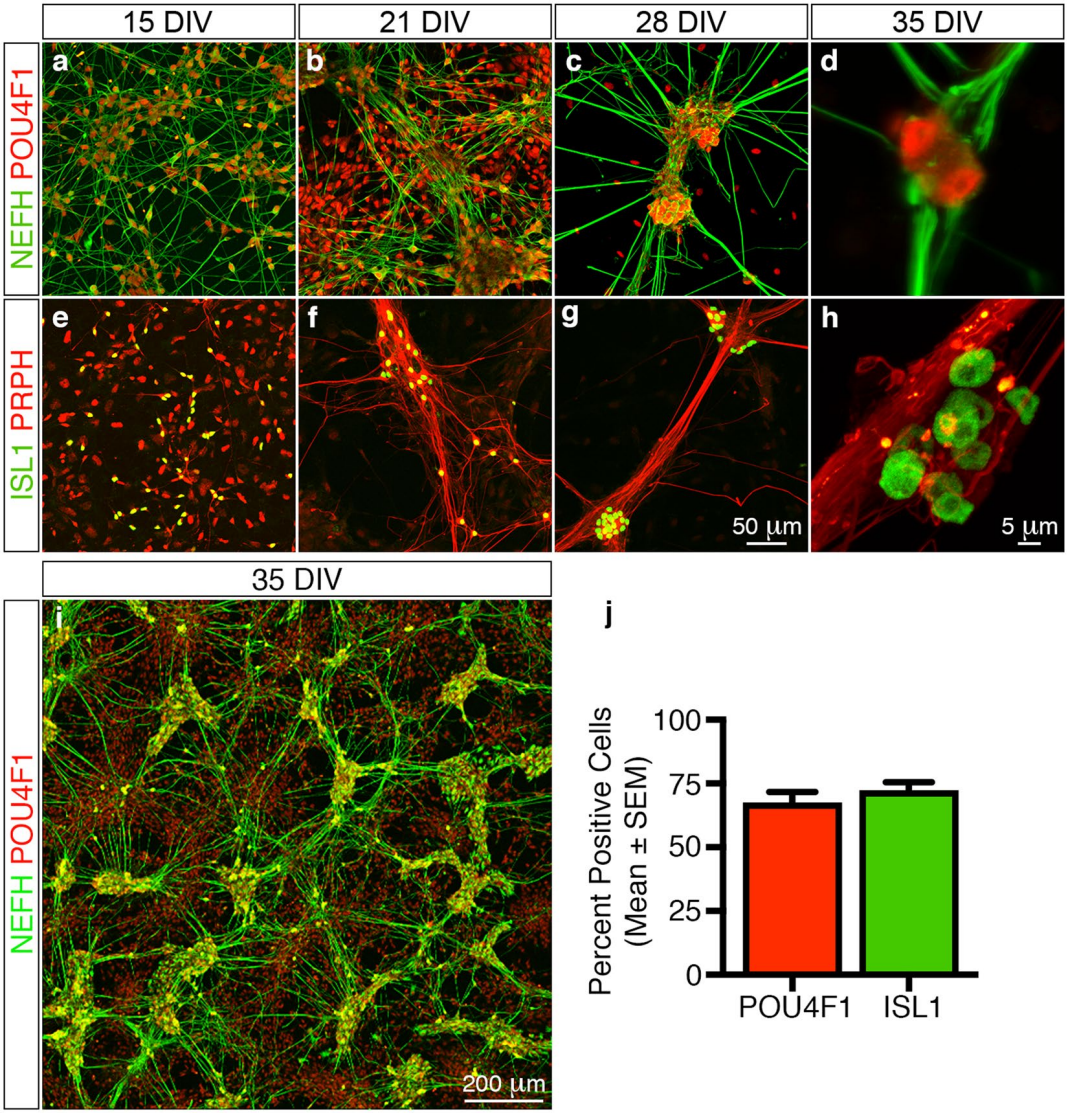
Molecular characterisation of *in vitro* differentiated peripheral sensory neurons. Time-course analyses using established marker combinations demonstrate that the differentiated cells exhibit molecular signatures that are similar to *in vivo* peripheral sensory neurons. (**a**–**d**) Numerous POU4F1^+^/NEFH^+^ neurons are apparent in the differentiation cultures 1 day after replating (**a**, 15 DIV). The neurons then coalesce over time into POU4F1^+^ neuronal ganglia that radially project NEFH^+^ axonal tracts (**b**–**d**, 21 to 35 DIV). (**e**–**h**) Similarly, numerous ISL1^+^/PRPH^+^ neurons are also readily apparent in the differentiation cultures 1 day after replating (**e**, 15 DIV). These in turn also coalesce into ISL1^+^ neuronal ganglia that radially project PRPH^+^ axonal bundles (**f**–**h**, 21 to 35 DIV). (**i**) Overview image illustrating the elaborate *in vitro* neuronal networks that are composed of distinct POU4F1^+^ neuronal ganglia and radially projecting NEFH^+^ axonal tracts formed at 35 DIV. (**j**) Quantitative analysis of the differentiated neuronal cultures at 35 DIV demonstrates that 68 ± 4% and 72 ± 3% (mean ± SEM) of all neuronal ganglia contain POU4F1^+^ and ISL1^+^ cells, respectively. All images and quantification analyses were performed on neurons derived from at least three independent differentiation experiments. Abbreviations: DIV, days *in vitro*. Scale bars: (**a**–**c**, **e**–**g**) 50 μm, (**d**, **h**) 5 μm; (*i*) 200 μm.

We next characterised the *in vitro* derived neurons using combinations of established molecular markers to determine whether the cells truly phenocopied *in vivo* peripheral sensory neurons. The homeodomain transcription factor POU class 4 homeobox 1 (POU4F1) and insulin gene enhancer protein (ISL1) are essential for peripheral sensory neuron development and survival^17,18^. Moreover, the intermediate neurofilament proteins peripherin (PRPH) and neurofilament heavy (NEFH) are selectively expressed in the PNS within nociceptors, mechanoreceptors and proprioceptors^19^. We therefore employed combinations of these markers to characterise the *in vitro* derived neurons. We observed numerous POU4F1^+^/NEFH^+^ and ISL1^+^/PRPH^+^ neurons after replating (15 DIV) (Fig. 2a,e) which organised themselves sequentially (21 and 28 DIV) (Fig. 2b,c,f,g) into POU4F1^+^ (68 ± 4%, n = 137/203) and ISL1^+^ (72 ± 3%, n = 205/283) neuronal ganglia with radially projecting NEFH^+^ and PRPH^+^ axonal bundles (35 DIV) (Fig. 2d,h–j).

**Figure 3:**
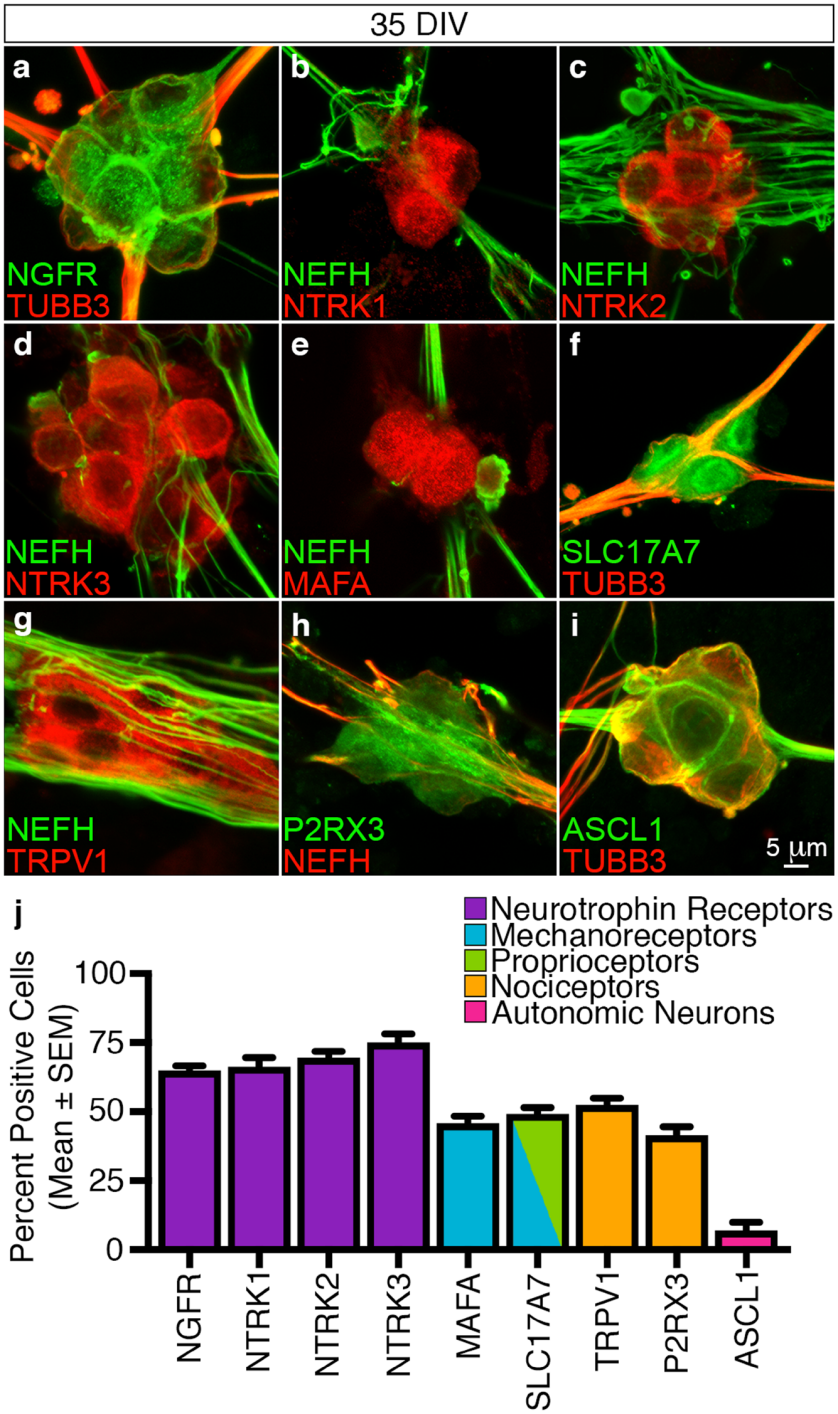
Molecular subtype characterisation of *in vitro* differentiated peripheral sensory neurons. Differentiated peripheral sensory neurons were characterised using combinations of subtype-specific markers at 35 DIV. (**a**–**d**) The differentiated peripheral sensory neurons express NGFR and all three NTRK family members (NTRK1, NTRK2 and NTRK3) thus confirming that the *in vitro* cultures are comprised of mechanoreceptive, nociceptive and proprioceptive subtypes. (**e**–**h**) Combinatorial expression of axonal (NEFH and TUBB3) with subtype-specific markers (MAFA, SC17A7, TRPV1 and P2RX3) demonstrates that the differentiated peripheral sensory neurons express marker permutations that are the molecular hallmarks of mechanoreceptors (NEFH^+^/MAFA^+^ and TUBB3^+^/SLC17A7^+^), proprioceptors (TUBB3^+^/SCL17A7^+^) and nociceptors (NEFH^+^/TRPV1^+^ and NEFH^+^/P2RX3^+^). (**i**) A small minority of neuronal ganglia were also determined to be ASCL1^+^ suggesting that some cells generated during the differentiation protocol were autonomic neurons. Additional overview images are presented in Supplementary Fig. 1. (**j**) Quantitative analyses (mean ± SEM) of neuronal ganglia at 35 DIV illustrate the molecular heterogeneity of the *in vitro* differentiated sensory neurons. All images and quantification analyses were performed on neurons derived from at least three independent differentiation experiments. Abbreviations: DIV, days *in vitro*. Scale bars: (**a**–**i**) 5 μm.

*In vivo* peripheral sensory neurons express the structurally distinct NGFR neurotrophin receptor and can be subdivided into mechanoreceptive, proprioceptive and nociceptive classes based on the selective combinatorial expression of NTRK family members (NTRK1, NTRK2 and NTRK3)^2^. We observed that the majority of the differentiated neuronal ganglia were NGFR^+^ (65 ± 2%, n = 130/201) (Fig. 3a,j) and expressed all three NTRK classes (NTRK1, 66 ± 3%, n = 141/214; NTRK2, 70 ± 2%, n = 124/182 and NTRK3, 75 ± 3%, n = 147/195) (Fig. 3b–d,j). Moreover, combinatorial expression analysis involving axonal (TUBB3 and NEFH) and sensory neuron subtype-specific markers including MAF bZIP transcription factor A (MAFA), solute carrier family 17 member 7 (SLC17A7), transient receptor potential vanilloid receptor-1 (TRPV1) and P2X purinoceptor 3 (P2RX3) demonstrated that the differentiated neurons also expressed marker permutations that are the molecular hallmarks of mechanoreceptive (NFL200^+^/MAFA^+^, 46 ± 3%, n = 110/235 and TUBB3^+^/SLC17A7^+^, 49 ± 2%, n = 90/185), proprioceptive (TUBB3^+^/SLC17A7^+^, 49 ± 2%, n = 90/185) and nociceptive (NEFH^+^/TRPV1^+^, 52 ± 2%, n = 100/189 and NEFH^+^/P2RX3^+^, 42 ± 3%, n = 108/263) neurons (Fig. 3e–h,j)^20–23^. Encouragingly, we also observed that only a low percentage of the neuronal ganglia were positive for the Achaete-scute homolog 1 protein (ASCL1^+^; 7 ± 3%, n = 35/407) (Fig. 3i,j) which demonstrated that a limited number of autonomic neurons were generated during the differentiation protocol^24^.

In summary, the *in vitro* derived neurons displayed morphological similarity and expressed specific molecular combinations of transcription factors, neurofilaments, membrane receptors and ion channels that were the hallmarks of all three classes of peripheral sensory neurons. We therefore concluded that differentiating hESCs using a combination of dual-SMAD inhibition and early WNT activation coupled with small-molecule inhibition of the Notch, VEGF, FGF and PDGF signaling pathways was a robust protocol for the generation of *in vitro* peripheral sensory neurons.

### Functional characterisation of differentiated peripheral sensory neurons

**Figure 4:**
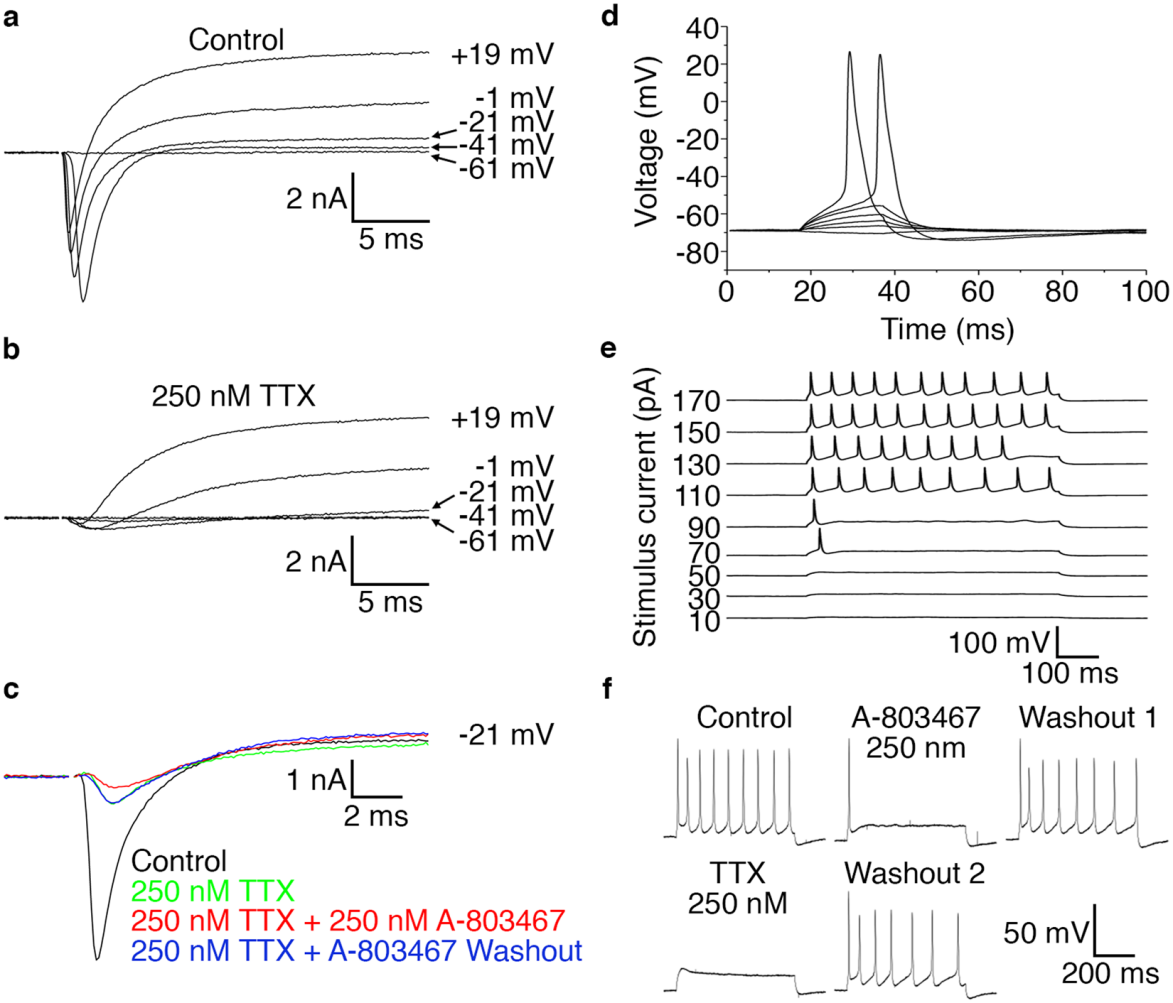
*In vitro* derived peripheral sensory neurons acquire electrophysiological properties. Whole-cell patch-clamp recordings taken from individual differentiated peripheral sensory neurons at 35 DIV. (**a**) Representative voltage-clamp recording showing voltage-gated Na^+^- and K^+^-like currents. Current responses to families of depolarising steps from a holding voltage of −71 mV to the levels indicated are superimposed. Large transient inward currents are observed at voltages ≥−41 mV. (**b**) Representative voltage-clamp recording following addition of TTX showing that a major fraction of the voltage-gated Na^+^ channels are blocked and that outward currents with characteristics of delayed rectifier K^+^ channels remain. Also note that small inward currents remain in the presence of TTX. Current responses to families of depolarising steps from a holding voltage of −71 mV to the levels indicated are superimposed. (**c**) Representative voltage-clamp recording showing both TTX-sensitive and TTX-resistant voltage-gated transient inward currents and that a TTX-resistant component is reversibly blocked by the application of the SCN10A-selective inhibitor A-803647. Current responses to voltage steps from −121 mV to −21 mV are shown. A holding voltage of −121 mV was used to minimise the inactivation of voltage-gated Na^+^ currents. (**d**) Representative sub-threshold voltage responses and action potentials evoked by 20-ms current steps from a holding current of 0 mV to levels ranging from −10 to + 110 pA in 20-pA increments. All-or-none action potentials are induced by the two largest stimuli. (**e**) Representative sub- and suprathreshold voltage responses and action potentials to 600 ms current steps of amplitudes as indicated. Repetitive action potential trains are observed at the larger stimuli. (**f**) Representative action potential train following a 600 ms stimulus current of 170 pA. Application of the SCN10A-selective inhibitor A-803647 to the same cell abolishes repetitive firing with only the first action potential remaining. This block is reversible and firing activity is restored upon washout. Subsequent application of TTX completely blocks all action potential generation with repetitive firing activity being recovered once again after TTX washout. All electrophysiology recordings were performed on neurons derived from at least three independent differentiation experiments. Abbreviations: DIV, days *in vitro*; ms, millisecond; mV, millivolt; pA, picoampere; TTX, tetrodotoxin.

The use of differentiated peripheral sensory neurons for studying PNS development and injury depends on the ability of the *in vitro* cells to faithfully recapitulate the functional properties of *in vivo* sensory neurons particularly in terms of their electrophysiological properties and potential for myelination^14,25,26^. We first addressed the electrophysiological modality by whole-cell patch-clamp recordings at 35 DIV (Fig. 4) since previous studies have demonstrated that neuronal maturation at the level of gene transcription has largely plateaued by this time point^8,14^. We focused our characterisation efforts on the nociceptive class of sensory neurons since this subtype exhibit features that are amenable for *in vitro* functional analyses^25^. One such attribute is the presence of tetrodotoxin (TTX) -resistant voltage-gated sodium channel alpha subunits 5 (SCN5A), 10 (SCN10A) and 11 (SCN11A) that are expressed in human nociceptive sensory neurons^27,28^.

The differentiated sensory neurons had a membrane resting potential of −62 ± 1 mV (n = 30), membrane time constant of 7.8 ± 0.9 ms (n = 22), input resistance of 174 ± 17 MΩ (n = 22) and a membrane capacitance of 48 ± 5 pF (n = 22). Depolarising voltage steps elicited fast initial transient inward currents at voltages of up to >0 mV and slower more sustained outward currents in all recorded cells (n = 24) (Fig. 4a). These observations are consistent with the opening of voltage-gated Na^+^ and K^+^ channels, respectively^29^. Application of 250 nM TTX blocked a major fraction of the initial transient voltage-gated currents in all recorded cells (94 ± 2%, n = 10) (Fig. 4b). However, a majority of the recorded neurons (n = 8) also displayed TTX-resistant currents that exhibited slow channel kinetics typically associated with SCN10A^7^. Moreover, a proportion of these TTX-resistant currents were blocked by the application of 250 nM of the SCN10A selective inhibitor A-803647 (53 ± 5%, n = 5/8) (Fig. 4c). We hypothesised that the remaining TTX- and A-803467-resistant currents (n = 3/8) were possibly be due to Na^+^ channels encoded by SCN11A^27^ or SCN5A^28^ respectively, but the relatively small amplitudes and simultaneous presence of outward currents precluded a definite identification.

To further test the electrophysiological functionality of the differentiated neurons we assessed their ability to fire action potentials. All recorded cells elicited action potentials in response to suprathreshold current injections (n = 27) (Fig. 4d). The action potentials as analysed for stimulus intensity >2 x threshold, showed a clearly positive peak (26 ± 3 mV) and a long duration (4.0 ± 0.4 ms at half-peak amplitude) (n = 16). Moreover, we observed that the majority of the neurons (n = 17/20) fired sustained action potential trains at higher prolonged current steps (Fig. 4e). Treatment of the cells with 250 nM A-803467 blocked this repetitive firing without affecting the first action potential in all cells tested (n = 11). Application of 250 nM TTX completely blocked all action potential generation in a majority of cells (n = 7/13) (Fig. 4f) but a subgroup of cells (n = 6/13) exhibited regenerative potentials in the presence of TTX. Taken together, our electrophysiological characterisation demonstrates that the *in vitro* differentiated neurons exhibit properties that are consistent with mature *bona fide* nociceptive sensory neurons. Moreover, our data suggests that SCN10A and possibly SCN11A and/or SCN5A are commonly expressed in our differentiated sensory neurons and that at least SCN10A contributes to repetitive firing of action potentials.

**Figure 5:**
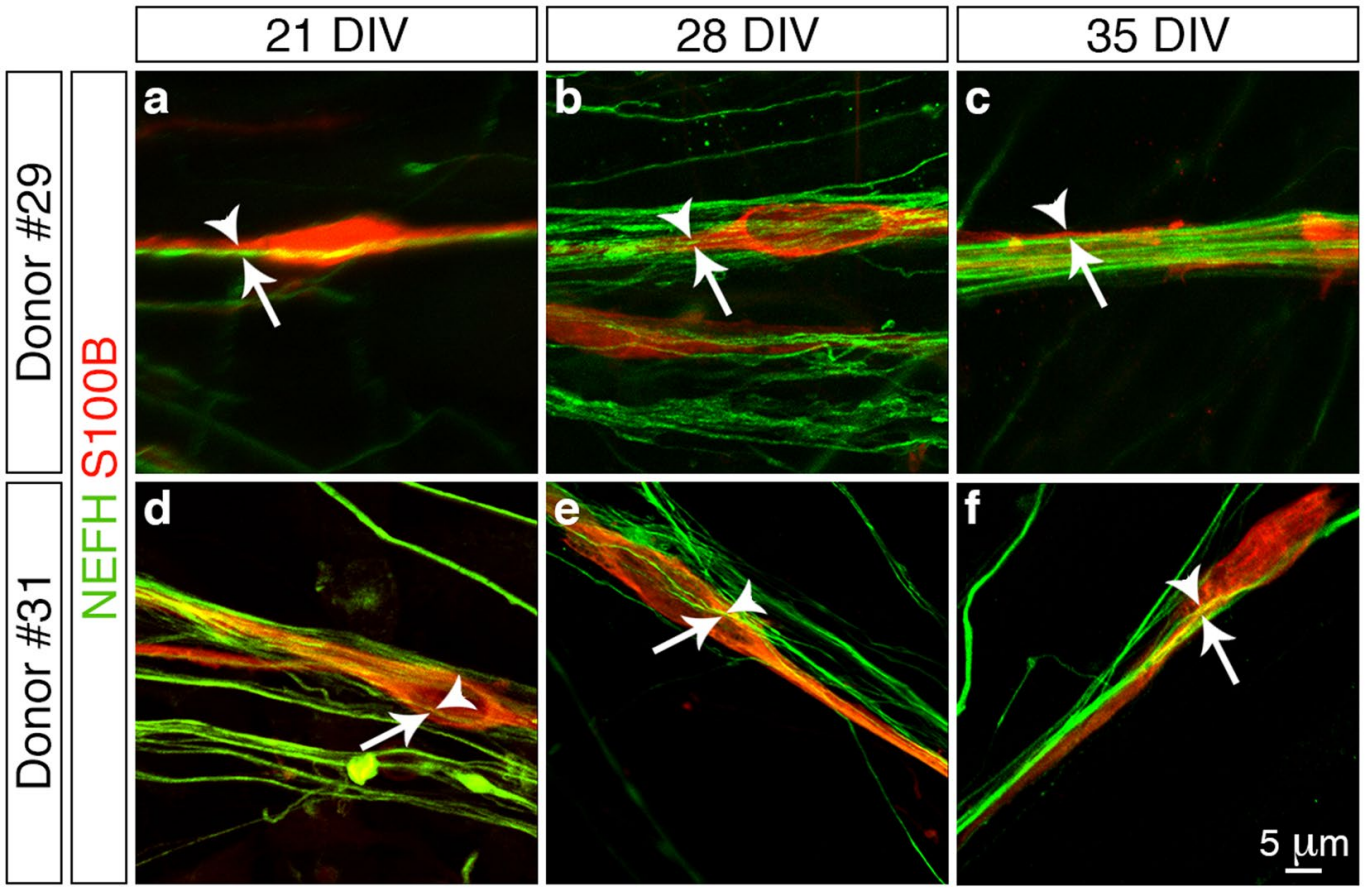
Association of *in vitro* derived peripheral sensory neurons and human Schwann cells. (**a**–**f**) Confocal z-stack reconstructions of donor Schwann cells (**a**–**c**, donor #29, 7 months old and **d**–**f**, donor #31, 15 months old) and *in vitro* differentiated peripheral sensory neuron co-cultures. Numerous S100B^+^ Schwann cells (arrowheads) align continuously with NEFH^+^ neurites (arrows) following one week of co-culture (**a** and **c**, 21 DIV) and over time the Schwann cells become intimately associated with the radiating axonal bundles (**b** and **e**, 28 DIV; **c** and **f**, 35 DIV). All images were derived from at least three independent co-culture experiments. Additional overview images are presented in Supplementary Fig. 2. Abbreviations: DIV, days *in vitro*. Scale bar: (**a**–**f**) 5μm.

**Figure 6:**
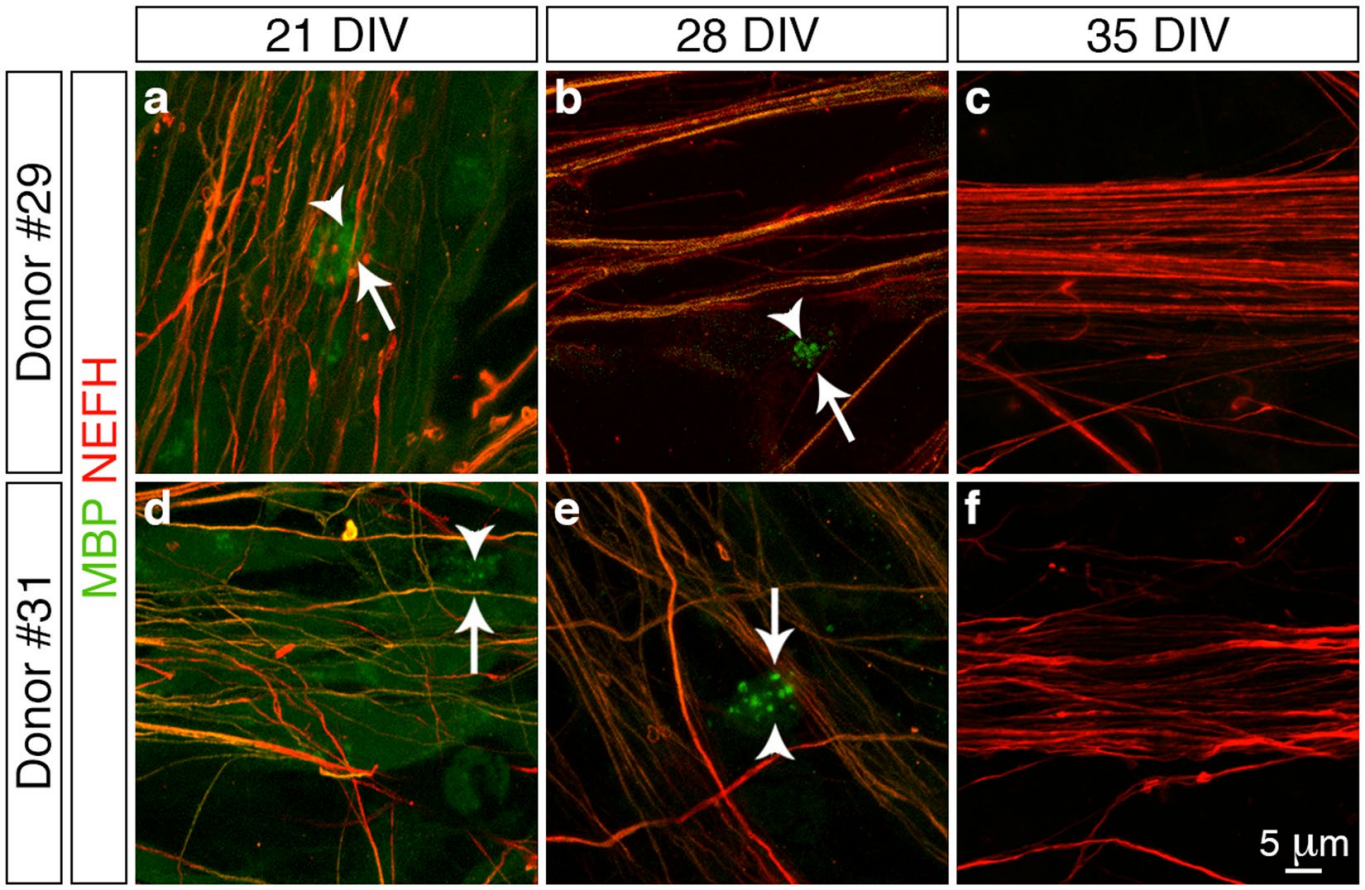
Myelination of *in vitro* derived peripheral sensory neurons by human Schwann cells. (**a**–**f**) Confocal z-stack reconstructions of donor Schwann cells (**a**–**c**, donor #29, 7 months old and (**d**–**f**), donor #31, 15 months old) and *in vitro* differentiated peripheral sensory neuron co-cultures. Schwann cells exhibiting MBP^+^ punctate foci (arrowheads) align continuously with NEFH^+^ neurites (arrows) following both one (**a** and **d**, 21 DIV) and two weeks (**b** and **e**, 28 DIV) of co-culture. However, no structural myelin segments were observed and MPB immunoreactivity was observed to rapidly decline during the third week of co-culture with no MBP^+^ Schwann cells being observed (**c** and **f**, 35 DIV). All images were derived from at least three independent co-culture experiments. Additional overview images are presented in Supplementary Fig. 3. Abbreviations: DIV, days *in vitro*. Scale bar: (**a**–**f**) 5 μm.

Next we addressed another functionality aspect of the *in vitro* derived peripheral sensory neurons by determining whether they could be myelinated by co-culture with human Schwann cells derived from two separate donor patients. We initially assessed whether the donor cells could associate with the peripheral sensory neurons by immunostaining for S100 calcium-binding protein B (S100B)^30^ in combination with NEFH to demarcate the donor cells and sensory neurons, respectively (Fig. 5). We observed that numerous S100B^+^ Schwann cells (arrowheads) aligned with the NEFH^+^ neurites (arrows) following one week of culture (21 DIV) (Fig. 5a,d) and that over time (28 and 35 DIV) the Schwann cells became intimately associated with the radiating axonal bundles (Fig. 5b,c,e,f). Since we observed this close association between the donor Schwann cells and the *in vitro* derived peripheral sensory neurons we next investigated whether this interaction triggered and supported the process of myelination. Myelin binding protein (MBP) is a major component of compact myelin sheaths^26^ and we therefore assessed for the presence of this integral protein in combination with NEFH to demarcate any myelinating Schwann cells that ensheathed the peripheral sensory neurons (Fig. 6). We detected numerous MBP^+^ Schwann cells (arrowheads) that were aligned with NEFH^+^ neurites (arrows) following both one (21 DIV, Fig. 6a,d) and two weeks (28 DIV, Fig. 6b,e) of co-culture. However, we observed that immunoreactivity for MBP appeared as distinct punctate foci and lacked organisation into structural myelin segments as has been documented for previous *in vitro* studies using cells derived from rodent models^31,32^. Furthermore, MBP immunoreactivity was observed to rapidly decline during the third week of co-culture and by 35 DIV no MBP^+^ Schwann cells were observed (Fig. 6c,f). In conclusion, co-culture of *in vitro* derived peripheral sensory neurons with human Schwann cells demonstrates that the donor cells align and intimately associate with the differentiated neurons. However, this interaction is not sufficient to trigger and support *in vitro* myelination under the culture conditions used in this present study.

### Assessing the efficacy of differentiated sensory neurons as an *in vitro* peripheral nerve injury model system

**Figure 7:**
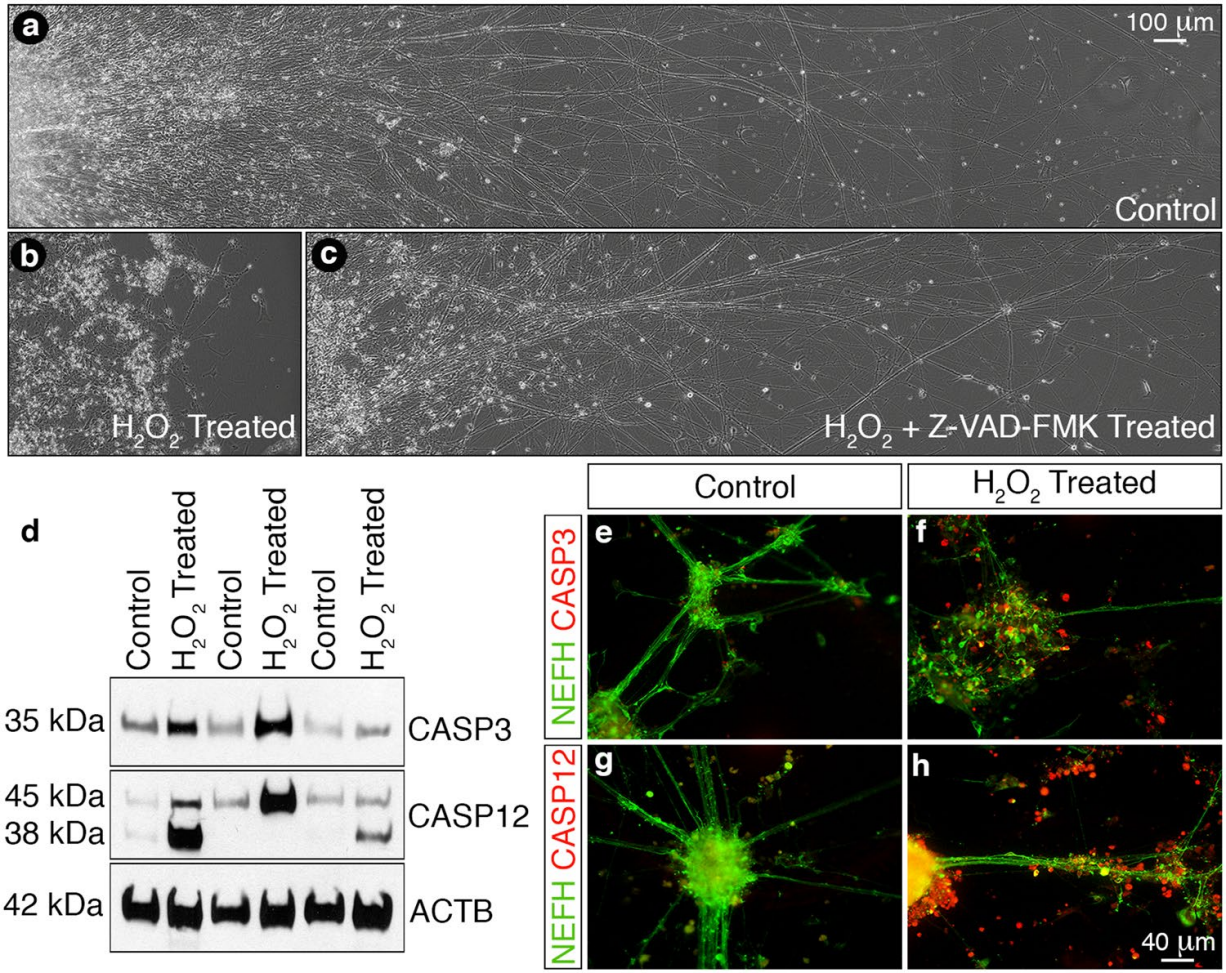
*In vitro* derived sensory neurons as a surrogate model for peripheral nerve injury. (**a**–**c**) Phase contrast images of control (**a**) and H_2_O_2_ treated (**b**,**c**) *in vitro* sensory neurons following 48 h culture in the absence (**b**) or presence (**c**) of the pan-caspase inhibitor Z-VAD-FMK. Exposure of the differentiated cultures to H_2_O_2_ induces neurite retraction (**b**) while inclusion of Z-VAD-FMK (**c**) blocks this process and the neuronal networks are largely preserved. (**d**) Immunoblotting of control and H_2_O_2_ treated *in vitro* sensory neuron cultures. Exposure of the cells to H_2_O_2_ for 24 h induces the upregulation of both CASP3 and CASP12. Also note the processed 38 kDa CASP12 fragment in two of the three cultures. ACTB was used as a loading control. The original full-length immunoblotting images are presented in Supplementary Fig. 4. (**e**–**h**) Immunocytochemical staining for CASP3 (**e**–**f**) and CASP12 (**g**–**h**) in control (e and g) and H_2_O_2_ treated (f and h) *in vitro* sensory neuron cultures. Numerous CASP3^+^ (**f**) and CASP12^+^ (**h**) cells are observed after exposure of the cultures to H_2_O_2_ for 24 h_._ Moreover, extensive disintegration of NEFH^+^ axon bundles (**f** and **h**) is also observed upon H_2_O_2_ treatment. Scale bars: (**a**–**c**) 100 µm, (**e**–**h**) 40 µm.

The use of differentiated peripheral sensory neurons for studying peripheral nerve injury (PNI) and disease depends on the ability of the *in vitro* cells to faithfully mimic the response of *in vivo* sensory neurons to physical or chemical insults^7^. To assess the usefulness of the *in vitro* derived cultures as a surrogate injury model we investigated the effects of hydrogen peroxide (H_2_O_2_) treatment on the differentiated neurons at 35 DIV (Fig. 7) since our previous study suggested that oxidative stress plays a key role in PNI cell death^33^. Treatment of the differentiated peripheral sensory neurons for 48 h with 1 mM H_2_O_2_ resulted in the disassembling of the neuronal ganglia coupled with widespread neurite retraction compared to control cultures (Fig. 7a,b). However, the neurite networks were largely preserved in the presence of the pan-caspase inhibitor Z-VAD-FMK (20 μM) (Fig. 7c) suggesting that apoptosis was the major intracellular process underlying the observed response to H_2_O_2_ treatment. To address this hypothesis we prepared protein extracts from control and H_2_O_2_ treated cells and observed an increase in the expression of both caspase-3 (CASP3) and caspase-12 (CASP12) in the H_2_O_2_ exposed neurons by immunoblotting (Fig. 7d). Moreover, this upregulation was confirmed by immunocytochemistry as we detected elevated levels of both active CASP3 and CASP12 in the H_2_O_2_ treated cultures (Fig. 7e–h). In conclusion, treatment of the *in vitro* peripheral sensory neurons with H_2_O_2_ results in oxidative stress-triggered apoptosis and mimics the molecular response observed in rodent models of PNI^33^. This proof-of-concept study therefore demonstrates the potential use of hESC-derived neurons as surrogate models for the study of PNS injury and disease.

## Discussion

**Figure 8:**
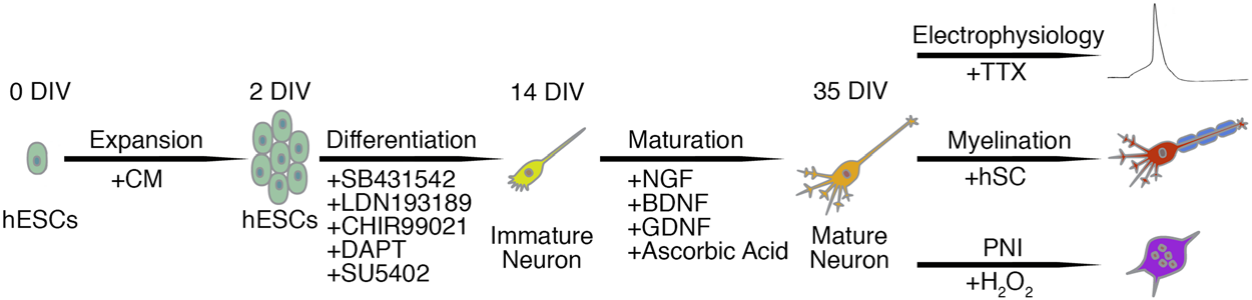
Schematic diagram of the *in vitro* peripheral sensory neuron model. hESCs were seeded as single-cell monolayers and expanded in conditioned medium to reach optimal confluence (0–2 DIV). The cells were then differentiated into naïve neurons by the use of small molecule inhibitors (2–14 DIV). The derived cells were then grown in a defined neurotrophic factor cocktail (14–35 DIV) to yield mature peripheral sensory neurons that were subsequently assessed for their electrophysiological properties and myelination amenability in addition to their suitability as an *in vitro* model system to study PNI. Abbreviations: CM, conditioned medium; DIV, days *in vitro*; hSC, human Schwann cells; PNI, peripheral nerve injury; TTX, tetrodotoxin.

This study demonstrates that the use of small-molecule inhibitors is a robust method for deriving peripheral sensory neurons from hESCs. The resulting electrically active neurons recapitulate several hallmarks of *bona fide* peripheral sensory neuron morphology and express the established spectrum of canonical- (POU4F1, ISL1) and subtype-specific (NTRK1, NTRK2, NTRK3) peripheral sensory neuron markers in comparable proportions. Moreover, the differentiated cells associate with donor-derived human Schwann cells and are also an amenable model system to investigate the molecular mechanisms underlying neuronal death following PNI (Fig. 8). Thus, the *in vitro* sensory neurons present an attractive and unlimited source of biological material for comparative studies that specifically address human peripheral sensory development, injury and disease.

The first unique aspect of this study is that reproducible numbers of all three peripheral sensory neuron subtypes were derived across multiple differentiation experiments. This presumably arises due to our experimental approach where the initial seeding of hESCs as single-cell monolayers promoted clonal expansion from single cells that resulted in a consistent and repeatable differentiation regime. These observations differ from those of previous studies where essentially the same differentiation protocol was employed for the derivation of variable numbers of *in vitro* peripheral sensory neuron subclasses or even cultures that were predominantly composed of NTRK1^+^ nociceptors^8,10,11,14^. Such experimental discrepancies highlight that user-specific differences exist in studies involving hESCs and demonstrate that several critical factors need to be considered when undertaking such experiments. Small differences in seeding conditions and pluripotency status of the hESC population prior to differentiation in addition to active inhibitor concentrations and timing regimes can have major influences on experimental outcome^34–36^. Notwithstanding, establishing the mechanisms by which *in vitro* peripheral sensory neurons establish modality specific identity is an important step for tailoring differentiation regimes to selectively enrich for specific sensory neuron subclasses. For example, the derivation of pure nociceptive neuronal populations would be particularly beneficial for studies that are focused on the development of pharmacological agents for the treatment of neuropathic and inflammatory pain^7^.

A central question raised by our study is whether the *in vitro* peripheral sensory neurons truly phenocopy their *in vivo* counterparts since they differentiate in an environment that is distinct from that of the developing nervous system^36^. However, our results do provide several independent lines of evidence that demonstrate that the vast majority of the differentiated cells display both the molecular and functional characteristics possessed by endogenous sensory neurons. For example, *in vivo* sensory neurons exhibit diversity in their expression of NTRKs^37^. We observed that the differentiated peripheral sensory neurons also displayed similar marker repertoires. Moreover, a consistent heterogeneous proportion of NTRK1^+^, NRTK2^+^ and NTRK3^+^ neurons were reproducibly generated across multiple differentiation experiments with greater enrichment for each subtype compared to a recent study^8^. This improvement suggests that seeding hESCs as single-cell monolayers is a superior differentiation approach since it apparently promotes stochastic NRTK receptor expression and implies that individual neural progenitors have an equivalent chance of differentiating into any of the NRTK subclasses under optimal culture conditions. Moreover, the derivation of all three sensory neuron NTRK^+^ subtypes suggests that the step-wise hierarchical modality of peripheral sensory neuron development is recapitulated in our study^2^. This is contrast to that seen in previous investigations where the predominant enrichment of NTRK1^+^ nociceptors suggested that the differentiation regimes were biased to recapitulate only certain aspects of sensory neuron development^10,11,14^. However, it must be noted that the overlapping expression patterns of NTRKs in various subtypes makes it difficult for us to determine the actual proportions of peripheral sensory neuron classes present within our hESC-derived cultures^19^.

The suitability of *in vitro* differentiated neurons as development and disease models is ultimately determined by their functionality. All of the *in vitro* neurons that we recorded from exhibited voltage-gated Na^+^ channels whose depolarization characteristics were remarkably similar to those of *in vivo* sensory neurons^29^. In addition, our functional data also demonstrated that the *in vitro* cultures were enriched in the SCN10A voltage-gated Na^+^ channel and exhibited the broad action potential morphology and repetitive firing patterns characteristic of peripheral nociceptors^7,27^. Taken together, our data implies that the *in vitro* cultures contain neurons that bear similar electrophysiological characteristics to *in vivo* human nociceptors. Moreover, given that we observed respectable numbers of both TRPV1^+^ and P2RX3^+^ neuronal ganglia in our *in vitro* cultures, an extended electrophysiological analysis including neuronal responses to noxious- and inflammatory-agonists such as capsaicin and α,β-methylene-ATP would be interesting^21,38^.

Myelination of peripheral neurons by Schwann cells is a critical event during PNS development and cooperative signaling between peripheral axons and myelinating glia is critical for maintenance of nervous system function. We attempted to develop an *in vitro* co-culture system using the hESC-derived sensory neurons and human donor Schwann cells to provide a unique model to study the factors that influence myelination as well as diseases associated with myelin sheath degradation. To our knowledge ours is the first documented use of a human-specific co-culture model as previous studies have addressed similar questions using rodent-specific^31,32^ or cross-species systems that involved combinations of either differentiated human peripheral sensory neurons and rodent-derived Schwann cells^39,40^ or embryonic rodent primary DRG cultures and differentiated human Schwann cells^41^. We observed that our novel co-culture system recapitulated the first sequential features of myelination as indicated by Schwann cell alignment and ensheathment of the extending axon, which again suggests that the hESC-derived peripheral sensory neurons are functionally active^40,41^. However, despite these encouraging initial steps we did not observe the subsequent development of structural myelin segments and other axoglial landmarks such as node of Ranvier formation as has been documented for rodent-specific co-culture models^31,32^. The reason for lack of myelination in our study is currently unknown since we recapitulated a culture environment that has been previously demonstrated to trigger *in vitro* myelination^31^. However, there could be several possibilities such as the *in vitro* sensory neurons may not have been mature enough to provide a microenvironment and contact dependent cues to support Schwann cell myelination. However, more likely is the notion that the glial cells themselves exhibited phenotypic instability in the *in vitro* environment and were incapable of myelinating the hESC-derived neurons in part due to the age of the donor patients^42^. Future studies could therefore be aimed at developing a human-specific co-culture system involving both *in vitro* derived peripheral sensory neurons and Schwann cells, since the routine differentiation of this myelinating glial cell type from hESCs has been extensively documented^43–45^.

Animal cell cultures have been widely used as a tool to address neuronal cell death and survival together with axon regeneration^6^. However, there may be species-specific differences in the response to injury so it is therefore pertinent to develop relevant human cell systems and assays to advance new therapeutic strategies^4^. In this study, we demonstrated that our *in vitro* culture system may provide a suitable model to investigate peripheral sensory neuronal cell death. Previous experimental studies have indicated that peripheral nerve axotomy induces DRG sensory neuron death and this observation is supported indirectly by our previous clinical studies showing significant loss of DRG volume in nerve-injured patients^46^. Oxidative stress has been suggested to be a mediator of this cell death since anti-oxidants such as N-acetyl cysteine (NAC) are neuroprotective^33^. Furthermore, NAC down-regulates CASP3 signaling in axotomised sensory neurons^47^. We observed that H_2_O_2_ treatment of the differentiated sensory neurons induced upregulation of both CASP3 and CASP12 with the morphological characteristics of apoptosis being blocked by the use of a pan-caspase inhibitor. Although there is extensive literature citing the role of CASP3 as an executioner of neuronal apoptosis, the role for CASP12 is less documented. Activation of CASP12 has been associated with the endoplasmic reticulum (ER) stress response^48^ and direct activation of ER-stress by tunicamycin treatment induces rat DRG neuronal apoptosis^49^. Our human model system can therefore be used to further elucidate the relevant apoptotic pathways involved in sensory neuron cell death following peripheral nerve injury or disease.

In conclusion, our data demonstrates that small-molecule inhibition of select signaling pathways provides a rapid methodology to derive peripheral sensory neurons from hESCs. This approach provides an attractive alternative to rodent models and in combination with recent advances in gene editing and reprogramming technologies can provide a versatile experimental toolbox for future studies addressing human PNS development^50,51^. In addition to its potential impact on providing insight into currently unidentified mechanisms governing neuronal differentiation, such approaches will also contribute to the development of novel translational medicine methodologies and drug screening platforms for the treatment of peripheral neuropathies^51,52^.

## Methods

### Ethical Statements

Fibroblast isolation from embryonic CF-1 mice was carried out in accordance to that approved by the Animal Review Board at the Court of Appeal of Northern Norrland in Umeå (DNR #22–15). Human Schwann cells were isolated from two donor patients undergoing elective surgery following informed consent from both individuals. Human Schwann cell isolation was carried out in accordance to that approved by the Local Ethical Committee for Clinical Research at Umeå University (DNR #03–425).

### Cell Culture

hESCs (H9, WA09, Passage 31–41) were grown on irradiated CF-1 mouse embryonic fibroblasts (MEFs) in DMEM/F12 supplemented with 20% (v/v) KnockOut™ Serum Replacement, 1 x non-essential amino acids, 100 mM glutamine, 0.1 mM β-mercaptoethanol, 1 x Penicillin/Streptomycin and 4 ng/ml bFGF. hESC colonies were clump passaged onto new CF-1 MEFs following collagenase IV treatment. Human Schwann cells were grown on poly-D-lysine coated flasks in DMEM supplemented with 10% (v/v) foetal bovine serum, 50 ng/ml NRG1β and 10 µM forskolin^53^. Schwann cells were passaged using trypsin and their purity was assessed by S100B immunostaining. Schwann cells between passage 3 and 5 were used for all co-culture experiments and their purity was approximately 80% with the remainder of the primary culture consisting of fibroblasts.

### Peripheral Sensory Neuron Differentiation

hESCs were seeded as single cells and grown in MEF conditioned medium to reach optimal confluence. Peripheral sensory neurons were subsequently differentiated from hESC monolayers by a combination of dual-SMAD inhibition (10 μM SB431542 and 100 nM LDN193189) and early WNT activation (3 μm CHIR99021) coupled with small-molecule inhibition of the Notch (10 μΜ DAPT) and VEGF/FGF/PDGF (5 μM SU5402) signaling pathways^16^. Following completion of the differentiation protocol at 14 DIV the immature neurons were dissociated into single cells by accutase treatment and replated as droplets containing between 20,000 to 100,000 cells onto the required matrigel-coated culture vessels and/or glass coverslips in N2 media^16^ supplemented with 20 ng/ml BDNF, 20 ng/ml GDNF, 50 ng/ml NGF, 200 μm ascorbic acid and 10 μM Y27632. Two days after replating the medium was aspirated and N2 medium containing mitomycin-C (1 μg/ml) was added and the cells incubated for 1 h. The neurons were then washed and fresh N2 medium supplemented as described above (without 10 μM Y17632) was added. The cultures were subsequently fed twice a week. Mouse laminin-1 (1 μg/ml) was also added weekly to maintain neuronal adhesion to the culture vessels and/or glass coverslips. A total of ten independent differentiation experiments were performed during this study.

### Immunocytochemistry

Cells seeded on matrigel-coated glass coverslips were fixed in 4% (w/v) PFA in PBS for 10 min on ice prior to being washed 3 × 5 min with PBST (PBS and 0.1% (v/v) Triton X-100). The cells were then blocked for 1 h at room temperature in 10% (v/v) foetal calf serum in PBST and then incubated overnight at 4 °C with the required primary antibodies diluted in 5% (v/v) foetal calf serum in PBST. The cells were subsequently washed 3 × 5 min with PBST and then incubated for 1 h at room temperature with 5 ng/ml DAPI and the required secondary antibodies (Molecular Probes, 1:500) diluted in 5% (v/v) FCS in PBST. The cells were then washed 3 × 5 min in PBST and mounted. A complete list of all primary and secondary antibodies and dilutions used in this study are given in Table 1.

**Table 1:** List of antibodies used in this study.

Official Name	Synonyms	Dilution	Isotype	Manufacturer	Product Number
ACTB	β-actin	1:1000	Mouse IgG_2b_K	Millipore	MAB1501R
ASCL1	MASH1	1:100	Mouse IgG_1_	BD Biosciences	556604
CASP12		1:500	Rabbit IgG	Abcam	ab62484
CASP3		1:1000	Rabbit IgG	Cell Signalling	9662 S
ISL1	ISLET1	1:1000	Mouse IgG_2b_	DSHB	39.4D5
MAFA		1:300	Rabbit IgG	Bethyl Labs	IHC00352
MAP2		1:100	Mouse IgG_1_	Millipore	MAB364
MBP		1:00	Rat IgG_2a_	Millipore	MAB386
NEFH	NFLH, NFL200	1:500	Mouse IgG_1_	Covance	SMI31R
NGFR	p75^NTR^, CD271	1:100	Mouse IgG_1_	ATS	ABN07
NTRK1	TRKA	1:100	Rabbit IgG	Alomone Labs	ANT018
NTRK2	TRKB	1:100	Rabbit IgG	Alomone Labs	ANT019
NTRK3	TRKC	1:100	Rabbit IgG	Alomone Labs	ANT020
P2RX3	P2X3	1:500	Guinea Pig IgG	Millipore	AB5896
POU4F1	BRN3A	1:500	Rabbit IgG	Millipore	AB5945
PRPH		1:1000	Rabbit IgG	Millipore	AB1530
S100B		1:2000	Rabbit IgG	Dako	Z0311
SLC17A7	vGLUT1	1:400	Mouse IgG_1_	Millipore	MAB5502
TRPV1		1:1000	Rabbit IgG	Agrisera AB	AS132701
TUBB3		1:1000	Rabbit IgG	Abcam	ab18207
Guinea Pig IgG^Alexa488^		1:500	Goat IgG	Molecular Probes	A11073
Mouse IgG^Alexa488^		1:500	Goat IgG	Molecular Probes	A11001
Mouse IgG^Alexa568^		1:500	Goat IgG	Molecular Probes	A11004
Rabbit IgG^Alexa568^		1:500	Goat IgG	Molecular Probes	A11011
Rat IgG^Alexa488^		1:500	Goat IgG	Molecular Probes	A11006

### Scanning Electron Microscopy

Peripheral sensory neuron cultures were fixed in 2.5% (w/v) glutaraldehyde in 0.1 M cacodylate buffer (pH 7.2) overnight at 4 °C. The samples were washed 3 × 5 min in 0.1 M cacodylate buffer (pH 7.2) and dehydrated in a critical point dryer. Dehydrated samples were attached onto aluminium mounts using carbon adhesive tape and coated with 5 nm gold/palladium. Section morphology was examined using a Zeiss Merlin field emission scanning electron microscope using a secondary electron detector at a beam accelerating voltage of 4 kV and probe current of 150 pA.

### **Electrophysiology**

Membrane voltage and current responses were recorded at room temperature using the whole-cell patch-clamp configuration and a Zeiss Axiovert 25 microscope^54^. Recording pipettes were pulled from borosilicate glass (GC150) and had a resistance of 3 to 4 MΩ when filled and immersed in the extracellular recording solution (see below). The pipette-filling solution contained: 107 mM CH_3_CO_2_K, 18 mM KCl, 6.0 mM NaCl, 0.9 mM CaCl_2_, 0.4 mM Na_2_-GTP, 5.0 mM Mg-ATP, 2.5 mM EGTA and 10 mM HEPES (pH 7.2), with a concentration of free Ca^2+^ calculated to 9.9 10^−8^ M. The extracellular solution contained: 137 mM NaCl, 5.0 mM KCl, 1.0 mM CaCl_2_, 1.2 mM MgCl_2_, 10 mM HEPES and 10 mM glucose (pH 7.4). TTX and A-803467 were applied using a gravity-fed fast perfusion system controlled by solenoid valves. The tip of an eight-barreled pipette used for perfusion was positioned 100 to 200 µm from the studied cell. The solution exchange time constant during whole-cell recording with cells resting on the chamber bottom was approximately 50 ms^55^. Signals were recorded using an Axopatch 200B amplifier, a Digidata 1200 interface and pClamp software. The signals were sampled at 2 to 5 kHz after low-pass filtering at 1 to 2 kHz (−3 dB). For voltage-gated currents, the capacitive and leak current components were subtracted from the total current on basis of scaled current responses to negative voltage steps. Liquid-junction potentials were calculated with the pClamp software and compensated for in the voltages given. All electrophysiology recordings were performed on neurons derived from three independent differentiation experiments.

### Peripheral Sensory Neuron and Schwann Cell Co-culture

Peripheral sensory neurons were differentiated from hESCs as described above. Immature neurons at 14 DIV were dissociated into single cells by accutase treatment and combined with trypsin-dissociated human donor Schwann cells. The co-culture was replated as droplets containing 100,000 neurons and 20,000 human Schwann cells (5:1 ratio) onto matrigel-coated culture vessels and/or glass coverslips in N2 media^16^ supplemented with 20 ng/ml BDNF, 20 ng/ml GDNF, 50 ng/ml NGF, 200 μm ascorbic acid, 50 ng/ml NRG1β, 10 µM forskolin and 10 μm Y27632. The co-cultures were subsequently fed twice a week with fresh N2 medium supplemented as described above (without 10 μM Y17632). Mouse laminin-1 (1 μg/ml) was also added weekly to maintain cell adhesion. Co-culture experiments were performed using human donor-derived Schwann cell cultures between passages 1–4.

### Oxidative Stress Assays

Peripheral sensory neurons were differentiated from hESCs as described above. Immature neurons at 14 DIV were dissociated into single cells by accutase treatment and replated as droplets containing 100,000 cells onto matrigel-coated 6-well plates and glass coverslips in N2 media^16^ supplemented with 20 ng/ml BDNF, 20 ng/ml GDNF, 50 ng/ml NGF, 200 μm ascorbic acid and 10 μM Y27632. Two days after replating the medium was aspirated and N2 medium containing mitomycin-C (1 μg/ml) was added and the cells incubated for 1 h. The neurons were then washed and fresh N2 medium supplemented as described above (without 10 μM Y17632) was added. The cultures were subsequently fed twice a week. Mouse laminin-1 (1 μg/ml) was also added weekly to maintain cell adhesion. Cells at 35 DIV were exposed to 1 mM H_2_O_2_ for 48 h in the presence or absence of the pan-caspase inhibitor Z-VAD-FMK (20 µM) and subsequently analysed. For immunoblotting, peripheral sensory neurons were scraped from the 6-well plates in RIPA buffer containing protease inhibitor cocktail (Complete Mini). The protein extracts were centrifuged for 5 min at 4°C and the concentration of the soluble fraction was measured using the DC Protein Assay Kit. Immunoblotting was performed on soluble protein extracts (20 μg) using 12% (w/v) SDS-polyacrylamide gels and nitrocellulose membranes as previously described^56^. Reactive proteins were visualized using the ECL Prime Western Blotting Detection reagent. The blots were then exposed to Kodak X-OMAT light sensitive film to obtain images that were scanned using Epsom Imaging software. Immunocytochemistry on peripheral sensory neurons seeded on glass coverslips was performed as described above. A complete list of all primary and secondary antibodies and dilutions used in this study are given in Table 1.

### Image Acquisition

All images were captured using a Zeiss LSM 710 confocal microscope or Nikon Eclipse E800 microscope fitted with a Nikon DS-Ri1 digital colour camera. All images were compiled using Fiji^57^, Adobe Photoshop and/or Adobe Illustrator.

### Molecular Marker Quantification

All quantification analyses were performed on neurons derived from three independent differentiation experiments. Twelve randomly chosen objective fields were sampled for each marker from each independent differentiation experiment. The percentage of marker^+^ to DAPI^+^ cells was subsequently quantified for each objective field and this data was pooled to derive the mean ± standard error of the mean (SEM) for each marker.

### Data Analyses

All data analyses were performed using Prism7 and are presented as the mean ± standard error of the mean (SEM). *n* refers to total neuron number derived from three independent differentiation experiments.

## Electronic supplementary material

Supplementary Information

## Data Availability

The raw datasets generated and analysed during this current study are available from the corresponding author upon request.
