# Supplementary material for: Development and validation of an in vitro model system to study peripheral sensory neuron development and injury
Source: Sci Rep. 2018 Oct 29;8:15961. doi: 10.1038/s41598-018-34280-3 (PMC6206093; doi:10.1038/s41598-018-34280-3)
Supplement: Supplementary file 1 — Supplementary Information [file 41598_2018_34280_MOESM1_ESM.pdf]

Supplementary Information.

Development and validation of an *in vitro* model system to study peripheral sensory neuron development and injury.

Iwan Jones, Tushar Devanand Yelhekar, Rebecca Wiberg, Paul J. Kingham, Staffan Johansson, Mikael Wiberg & Leif Carlsson.

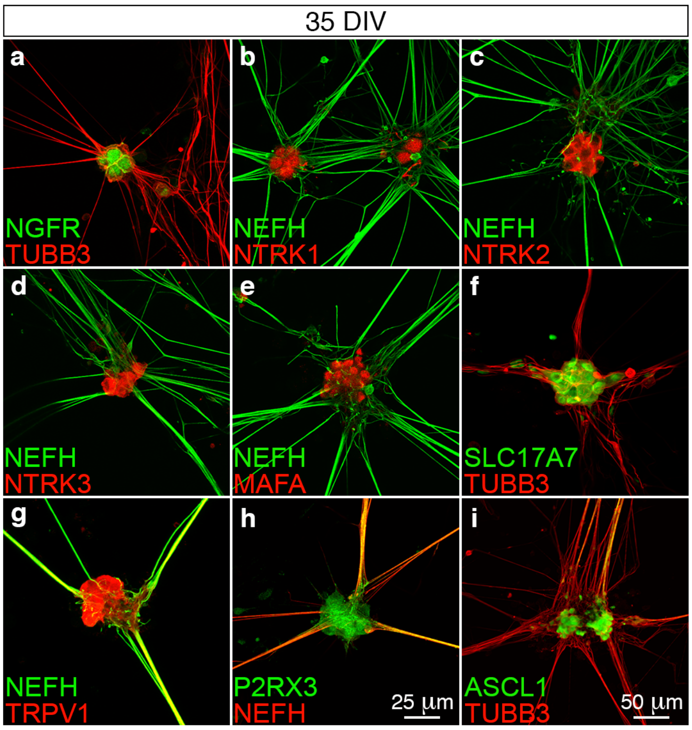

Supplementary Figure 1. Molecular subtype characterisation of differentiated peripheral sensory neurons. *In vitro* peripheral sensory neurons were characterised using subtype-specific markers. (a – d) The differentiated neurons express NGFR and all three NTRK members (NTRK1, NTRK2 and NTRK3) thus confirming that the *in vitro* cultures are comprised of mechanoreceptive, nociceptive and proprioceptive subclasses. (e – h) Combinatorial expression of axonal (NEFH and TUBB3) with subtype-specific markers (MAFA, SC17A7, TRPV1 and P2RX3) demonstrates that the differentiated neurons express marker permutations that are the hallmarks of mechanoreceptors ($NEFH^{+}$/$MAFA^{+}$ and $TUBB3^{+}$/$SLC17A7^{+}$), proprioceptors ($TUBB3^{+}$/$SCL17A7^{+}$) and nociceptors ($NEFH^{+}$/$TRPV1^{+}$ and $NEFH^{+}$/$P2RX3^{+}$). (i) A few neuronal ganglia were also $ASCL1^{+}$ suggesting that some cells were autonomic neurons. This supplementary figure accompanies the higher power images of *in vitro* sensory neuron subtype characterisation presented in Figure 3 of the manuscript. Abbreviations: DIV, days *in vitro*. Scale bar: (a – h) 25 µm; (i) 50 µm.

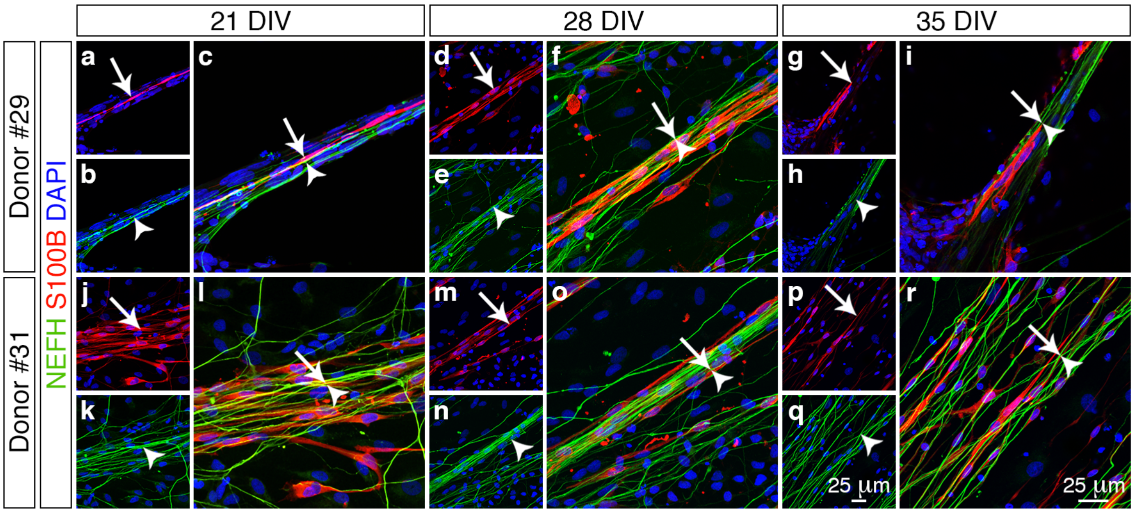

Supplementary Figure 2. Association of *in vitro* sensory neurons with human Schwann cells. (a – r) Confocal reconstructions of Schwann cells (a – i, donor #29 and j – r, donor #31) and differentiated peripheral sensory neuron co-cultures. Numerous S100B$^+$ Schwann cells (arrowheads) align with NEFH$^+$ neurites (arrows) following one week of co-culture (a – c and j – l, 21 DIV) and over time the Schwann cells become associated with the radiating axonal bundles (d – f and m – o, 28 DIV and g – i and p – r, 35 DIV). The unstained DAPI$^+$ nuclei are fibroblasts originating from the human Schwann cell cultures. This supplementary figure accompanies the higher power images of *in vitro* sensory neuron and Schwann cell association presented in Figure 5 of the manuscript. Abbreviations: DIV, days *in vitro*. Scale bars: (a – b, d – e, g – h, j – k, m – n, p – q) 25 µm, (c, f, i, l, o, r) 25µm.

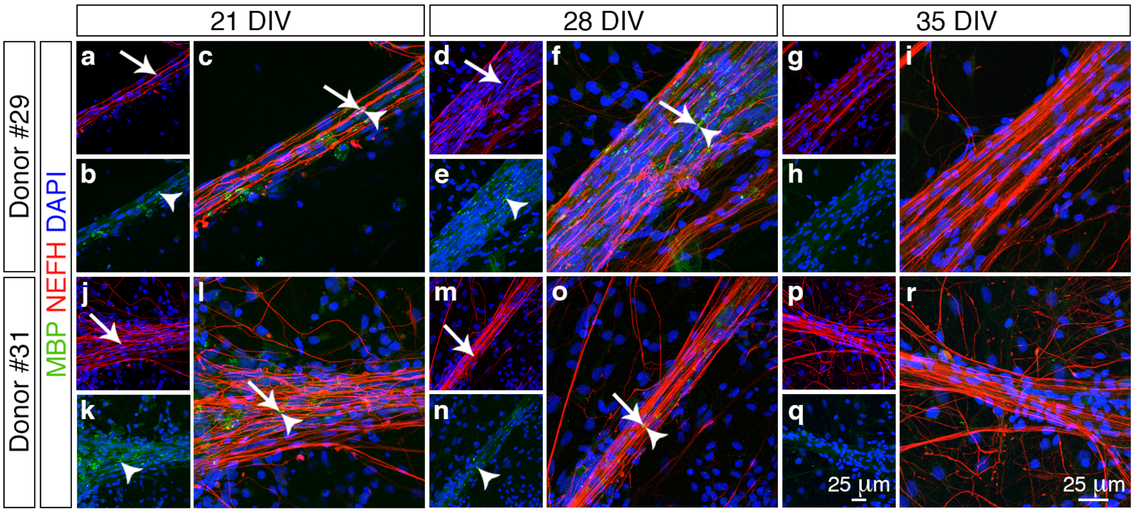

Supplementary Figure 3. Myelination of *in vitro* sensory neurons by human Schwann cells. (a – r) Confocal reconstructions of donor Schwann cells (a – i, donor #29 and j – r, donor #31) and differentiated peripheral sensory neuron co-cultures. Schwann cells exhibiting MBP^+ punctate foci (arrowheads) align continuously with $NEFH^+$ neurites (arrows) following both one (a – c and j – l, 21 DIV) and two weeks (d – f and m – o, 28 DIV) of co-culture. However, MPB immunoreactivity was observed to rapidly decline during the third week of co-culture (g – i and p – r, 35 DIV). The unstained $DAPI^+$ nuclei are fibroblasts originating from the human Schwann cell cultures. This supplementary figure accompanies the higher power myelination images presented in Figure 6 of the manuscript. Abbreviations: DIV, days *in vitro*. Scale bars: (a – b, d – e, g – h, j – k, m – n, p – q) 25 µm, (c, f, i, l, o, r) 25µm.

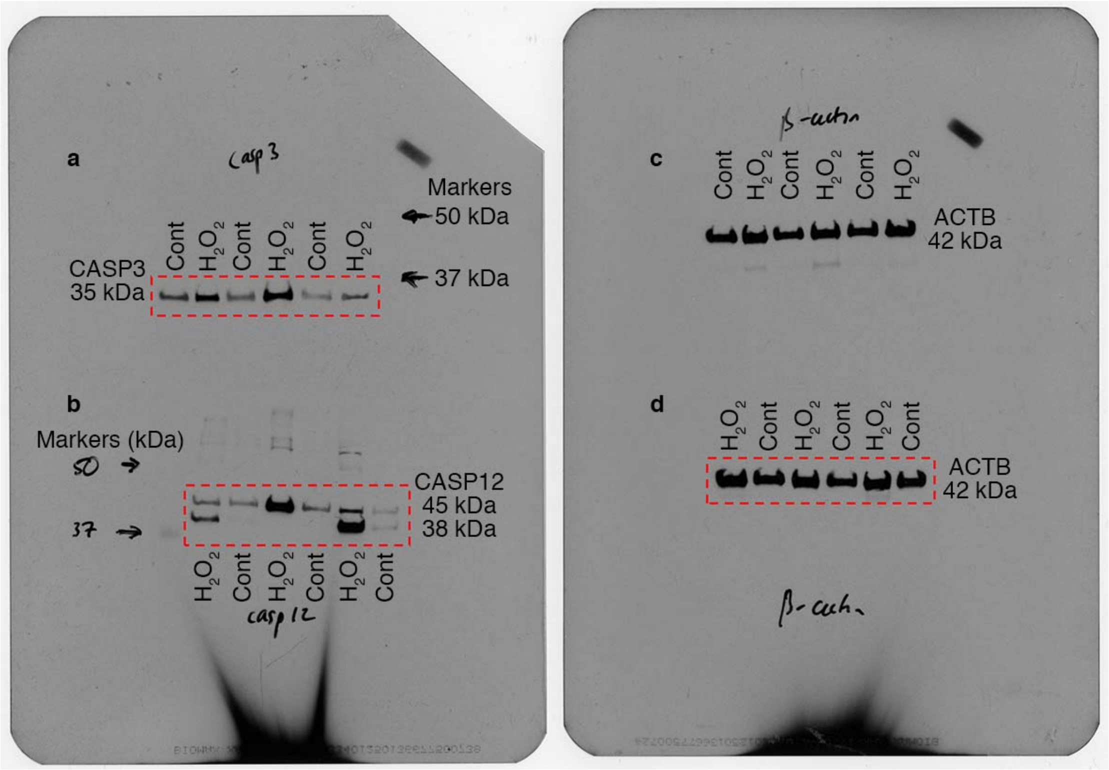

Supplementary Figure 4. Differentiated sensory neurons as an *in vitro* model for peripheral nerve injury. Scans of the original full-length immunoblotting images from control and H_2O_2 treated *in vitro* sensory neuron cultures. (a) CASP3, (b) CASP12, (c) ACTB for the CASP3 and (d) ACTB for CASP12. The red dashed boxes represent the approximate areas used to produce the cropped images presented in Figure 7d. Please note that the cropped CASP12 and ACTB images in Figure 7d are reversed from that presented here.
